# The oral and conjunctival microbiotas in cats with and without feline immunodeficiency virus infection

**DOI:** 10.1186/s13567-014-0140-5

**Published:** 2015-03-03

**Authors:** Scott J Weese, Jamieson Nichols, Mohammad Jalali, Annette Litster

**Affiliations:** Department of Pathobiology, University of Guelph, Guelph, Canada; Department of Veterinary Clinical Sciences, Purdue University, 625 Harrison St., West Lafayette, IN 47907 USA

## Abstract

The oral and conjunctival microbiotas likely play important roles in protection from opportunistic infections, while also being the source of potential pathogens. Yet, there has been limited investigation in cats, and the impact of comorbidities such as feline immunodeficiency virus (FIV) infection has not been reported. Oral and conjunctival swabs were collected from cats with FIV infection and FIV-uninfected controls, and subjected to 16S rRNA gene (V4) PCR and next generation sequencing. 9,249 OTUs were identified from conjunctival swabs, yet the most common 20 (0.22%) OTUs accounted for 76% of sequences. The two most abundant OTUs both belonged to *Staphylococcus*, and accounted for 37% of sequences. Cats with FIV infection had significantly lower relative abundances of Verrucomicrobia, Fibrobacteres, Spirochaetes, Bacteroidetes and Tenericutes, and a higher relative abundance of Deinococcus-Thermus. There were significant differences in both community membership (*P* = 0.006) and community structure (*P* = 0.02) between FIV-infected and FIV-uninfected cats. FIV-infected cats had significantly higher relative abundances of Fusobacteria and Actinobacteria in the oral cavity, and significantly higher relative abundances of several bacterial classes including Fusobacteria (0.022 vs 0.007, *P* = 0.006), Actinobacteria (0.017 vs 0.003, *P* = 0.003), Sphingobacteria (0.00015 vs 0.00003, *P* = 0.0013) and Flavobacteria (0.0073 vs 0.0034, *P* = 0.030). The feline conjunctival and oral microbiotas are complex polymicrobial communities but dominated by a limited number of genera. There is an apparent impact of FIV infection on various components of the microbiota, and assessment of the clinical relevance of these alterations in required.

## Introduction

The body harbours vast microbial communities (microbiotas) at different body sites [1]. The microbiota interacts closely with the immune system and alterations in the immune status of the host can result in changes in the microbiota, as has been shown with the fecal, anal and oral microbiotas of humans with human immunodeficiency virus (HIV) infection [2-5]. Feline immunodeficiency virus (FIV), another lentivirus, is disseminated in the cat population worldwide [6-10]. As with HIV in humans, FIV infection can result in progressive immune dysfunction, with a corresponding increase in a variety of infectious disease risks. A variety of ocular and oral manifestations of HIV infection can occur in humans, including both infectious and non-infectious causes [11], and it is possible that alterations in the local microbiota play a role in the pathophysiology of some of these diseases. Further, the oral microbiota in humans has been shown to have an influence on various infectious diseases, including both oral (e.g. gingivitis, stomatitis, periodontitis) and extra-oral (e.g. infective endocarditis) disease, and alterations of the oral microbiota have been identified in individuals with HIV infection [2,12-14].

Understanding of the normal ocular and oral microbiotas, and changes that occur in response to FIV infection, is important for understanding the impact of FIV infection and the pathophysiology of FIV-associated opportunistic infections. The objectives of this study were to describe the conjunctival and oral bacterial microbiotas and to evaluate the impact of FIV infection.

## Materials and methods

### Study population

Thirty-three cats were enrolled from a broader 5-year longitudinal controlled study investigating the clinical effects of FIV in naturally acquired infection. FIV infection status at the time of enrollment was determined by FIV antibody detection^a^ and later confirmed using polymerase chain reaction (PCR) for FIV RNA^b^ or virus isolation^c^. Of the enrolled cats, 19 were FIV-infected and 14 were FIV-uninfected. The cats enrolled in this study represent a convenience sample based on the timing of examination and sample collection for the larger study. FIV-infected cats were sourced from either a feline sanctuary (*n* = 15) or were privately owned (*n* = 4). FIV-uninfected cats were sourced from either the same feline sanctuary (*n* = 2) or were privately owned (*n* = 12). FIV-infected and uninfected cats were housed indoors and comingled within the sanctuary. Two of the privately owned cats, one FIV-infected and one FIV-uninfected, were allowed outdoors. None of the privately owned FIV-uninfected cats in this study were housed with FIV-infected cats. At the time of examination, all cats were re-tested for FIV antibodies.

Physical examination was performed at the time of sampling. Oral and conjunctival swab samples were collected with a sterile Dacron swab from the oral cavity or conjunctival surface, respectively. Oral health and gingival score were determined by visual inspection at the time of examination. The presence of gingivitis, stomatitis, gum recession, tooth root or furcation exposure, purulent material at the gumline, loose teeth or masses was considered evidence of dental disease. The gingival score was determined using the following scale: 0 – normal gingiva with sharp, non-inflamed edges; 1 – marginal gingivitis, minimal inflammation at free margin, no bleeding when pressure applied to the gingiva; 2 – moderate gingivitis, wider of inflammation at gingival margin, bleeding when pressure applied to gingiva; 3 – marked gingivitis, severe inflammation, bleeding can be present or absent when pressure applied to gingiva [15]. As cats were conscious during examinations, probing depths were not assessed and intraoral radiography was not performed.

The study was approved by the Purdue University Animal Care and Use Committee (protocol 1201000568).

### Sequencing

DNA extraction was performed using a commercial kit^d^. The V4 region of the bacterial 16S rRNA gene was then amplified using the primers S-D-Bact-0564-a-S-15 (5′-AYTGGGYDTAAAGNG-3′) and S-D-Bact-0785-b-A-18 (5′-TACNVGGGTATCTAATCC-3′) [16]. Sequencing was performed using an Illumina MiSeq^e^ and 2×250 chemistry.

### Data analysis

Mothur v1.33.3 [17] was used to assemble paired end reads and perform downstream analysis. After assembly, sequence files were depleted of sequences that were not consistent with the target amplicon size (240 bp), contained any ambiguous base calls or long runs (>8 bp) of holopolymers, or did not align with the correct 16S rRNA gene region. After alignment with the Silva 16S rRNA reference database [18], chimeras were detected using UCHIME [19] and removed. Taxonomy was then assigned using the RDP taxonomy database [20] and any non-bacterial sequences that remained were removed. Sequences were then binned into operational taxon units (OTUs) at a 3% dissimilarity level.

Wilcoxon signed rank tests were used to compare relative abundances of different phylogenetic groups. Subsampling was performed to normalize sequence number for subsequent analyses [21] through random selection of a number of sequences that was chosen to optimize both sequence numbers and the number of samples that could be included. Good’s coverage, diversity (inverse Simpson’s index), evenness (Shannon’s evenness index) and richness (Catchall) [22] were calculated and compared between groups using Wilcoxon signed rank test. Dendrograms were developed based on the Yue and Clayton measure of dissimilarity (a measure of community structure, which considers shared OTUs and their relative abundances) and traditional Jaccard index (a measure of community membership, which just considers the number of shared OTUs, not their abundance) and UniFrac [23] and analysis of molecular variance (AMOVA) were applied to compare microbial communities. Analysis of molecular variance was also used to compare communities. Principal coordinate analysis (PCoA) and indicator analysis [24] were also performed. A *P* value of ≤ 0.05 was considered significant for all analyses.

## Results

Cats ranged in age from 5 to 12 years of age (median 7 years). There was no difference in median age of the two groups (FIV-uninfected mean 7.2y, median 8y, range 5-10y; FIV-infected mean 7.3y, median 7y, range 5-12y) (*P* = 0.90). Four (31%) of the FIV-uninfected cats were neutered males and 9 (69%) were spayed females, while 12 (63%) cats in the FIV-infected group were neutered males and seven (37%) were spayed females (*P* = 0.15). Four (21%) of the FIV-infected cats were subjectively considered clinically healthy on a general physical examination, while the others had varying degrees of oral, dental, respiratory, skin or ocular disease. Six (46%) of FIV-uninfected cats were clinically normal, with the remaining cats having varying degrees of oral and skin disease. There was no difference in health status between groups (*P* = 0.24).

### Conjunctival microbiota

A total of 747 559 sequences from 32 cats (19 FIV infected, 13 FIV uninfected) passed all screening tests, with sequence numbers ranging from 956 to 53 810 per sample (mean 23 361, median 21 267). A total of 9249 OTUs was identified, yet the most common 20 (0.22%) OTUs accounted for 76% of sequences. The two most abundant OTUs both belonged to *Staphylococcus*, and accounted for 37% of sequences.

Alpha and beta diversity were analysed using a subsampled population of 3422 sequences per sample. This resulted in the exclusion of samples from three cats (two FIV-uninfected, one FIV-infected) that repeatedly yielded low sequence numbers. Alpha diversity data are presented in Table 1. Cats with FIV infection had significantly lower relative abundances of Verrucomicrobia, Fibrobacteres, Spirochaetes, Bacteroidetes and Tenericutes, and a higher relative abundance of Deinococcus-Thermus (Table 2). Predominant families and genera among the FIV-infected and FIV-uninfected cats are presented in Table 3. Significant differences in the relative abundance of genera accounting for at least 0.1% of sequences are presented in Table 4.

**Table 1:** **Alpha diversity comparison (medians) of the conjunctival microbiota of FIV-infected (** ***n*** **= 18) and FIV-uninfected (** ***n*** **= 11) cats**

	**FIV +**	**FIV -**	***P*** **value**
Diversity (inverse Simpson’s)	3.1	4.1	0.053
Evenness (Shannon’s evenness)	0.38	0.51	0.022
Richness (Catchall)	1022	1079	0.74
Coverage (Good’s coverage)	0.98	0.98	0.61

**Table 2:** **Phyla identified in the conjunctival microbiota of FIV-infected (** ***n*** **= 19) and FIV-uninfected (** ***n*** **= 13) cats**

**Phylum**	**FIV-infected**	**FIV-uninfected**	***P*** **value**
Firmicutes	0.55	0.43	0.20
Proteobacteria	0.37	0.30	0.37
Actinobacteria	0.0078	0.0057	0.50
Verrucomicrobia	0.0064	0.023	0.0014
Fibrobacteres	0.0056	0.024	0.019
Spirochaetes	0.0010	0.0082	0.026
Bacteroidetes	0.0041	0.012	0.0096
Chlamydiae	0.0015	0.0015	0.54
Fusobacteria	0.00099	0.0015	0.35
Tenericutes	0.00032	0.0022	0.0005
Deinococcus-Thermus	0.00031	0.000048	0.0083
TM7	0.00017	0.00032	0.89
Acidobacteria	0.000090	0.000057	0.53
Synergistetes	0.000038	0.000059	0.34
SR1	0.000033	0.00023	0.52
Deferribacteres	0.0000067	0	0.23
Lentisphaerae	0.0000048	0.00016	0.13
Elusimicrobia	0.0000010	0.00011	0.31
Armatimonadetes	0	0.000005	0.23

**Table 3:** **Predominant families and genera in the conjunctival microbiota of FIV-infected (** ***n*** **= 19) and FIV-uninfected (** ***n*** **= 13) cats**

**Family (relative abundance)**		**Genus (relative abundance)**	
**FIV +**	**FIV -**	**FIV +**	**FIV -**
Staphylococcaceae (0.45)	Staphylococcaceae (0.27)	*Staphylococcus* (0.42)	*Staphylococcus* (0.14)
Pseudomonadaceae (0.14)	Moraxellaceae (0.20)	*Pseudomonas* (0.11)	*Acinetobacter* (0.15)
Enterobacteriaceae (0.10)	Pseudomonadaceae (0.13)	Unclassified Enterobacteriaceae (0.090)	*Pseudomonas* (0.12)
Planococcaceae (0.062)	Aerococcaceae (0.54)	*Sporosarcina* (0.052)	Unclassified Clostridiales (0.058)
Unclassified Clostridiales (0.022)	Unclassified Clostridiales (0.028)	Unclassified Pseudomonadaceae (0.038)	*Psychrobacter* (0.055)
Unclassified Bacillales (0.020)	Bacillales incertae sedis XII (0.027)	Unclassified Clostridiales (0.022)	*Aerococcus* (0.054)
Unclassified Firmicutes (0.017)	Lachnospiraceae (0.022)	Unclassified Bacillales (0.017)	Unclassified Firmicutes (0.030)

**Table 4:** **Relative abundance of genera accounting for >0.1% of the conjunctival microbiota that were significantly different in FIV-infected (** ***n*** **= 19) and FIV-uninfected (** ***n*** **= 13) cats**

**Genus**	**FIV infected**	**FIV uninfected**	***P*** **value**
*Staphylococcus*	0.42	0.14	0.0026
Unclassified bacterium	0.051	0.19	0.0038
Unclassified Clostridiales	0.016	0.058	0.0029
Unclassified Pseudomonadaceae	0.038	0.0072	0.0096
Unclassified Firmicutes	0.011	0.030	0.0077
Unclassified Lachnospiraceae	0.010	0.036	0.0023
*Fibrobacter*	0.0056	0.024	0.019
5 genus incertae sedis	0.0046	0.019	0.0007
Unclassified Staphylococcaceae	0.0077	0.0010	0.0003
Unclassified Ruminococcaceae	0.0034	0.014	0.0043
Unclassified Bacteroidetes	0.0015	0.010	0.0017
Unclassified Clostridia	0.0017	0.0049	0.0018
*Treponema*	0.00089	0.0076	0.021
*Phenylobacterium*	0.0034	0.00038	0.0024

**Figure 1:**
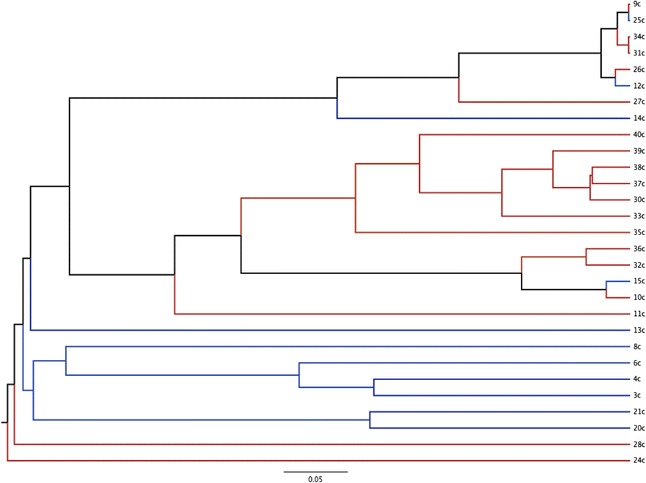
**Bacterial community structure of the conjunctival microbiota of FIV-infected and FIV-uninfected cats.** Dendrogram based on the Yue and Clayton index of dissimilarity characterizing the microbial population structure of the conjunctiva in 18 cats with FIV infection (red) and 11 uninfected (blue) cats.

**Figure 2:**
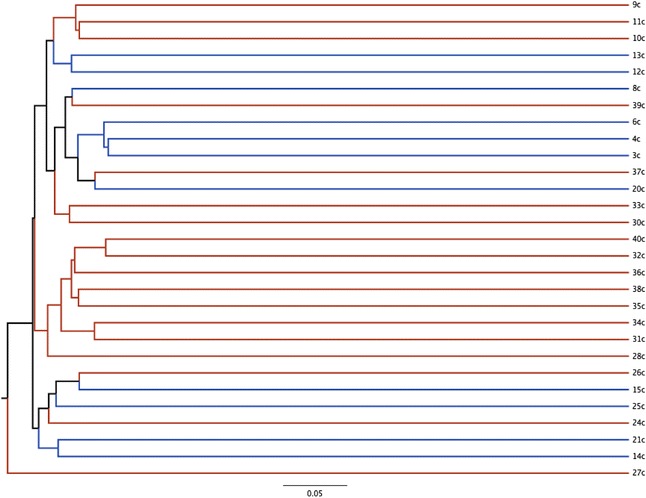
**Bacterial community membership of the conjunctival microbiota of FIV-infected and FIV-uninfected cats.** Dendrogram based on the classical Jaccard index demonstrating the community membership (operational taxon units that are present, irrespective of relative abundance) of 18 cats with FIV infection and 11 uninfected cats.

**Figure 3:**
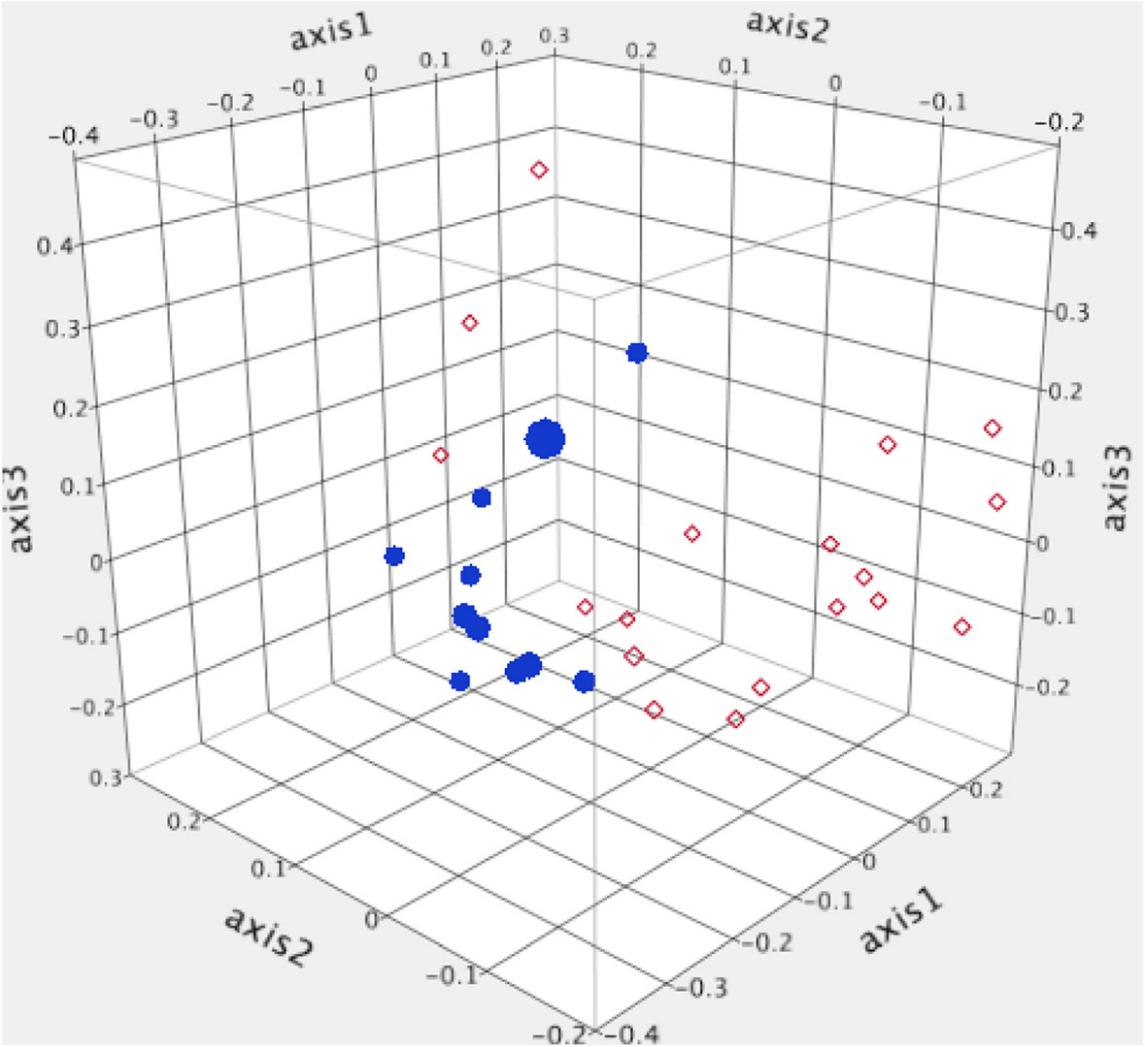
**Principle coordinate analysis of the community membership of cats with FIV infection and uninfected controls.** Three dimension principle coordinate analysis plot based on the classical Jaccard index depicting the community membership of the conjunctival microbiota of FIV-infected (n = 18, red diamonds) and FIV-uninfected (*n* = 11, blue dots) cats.

Dendrograms based on the Yue and Clayton (community structure) and Jaccard (community membership) indices are displayed in Figures 1 and 2. There were significant differences in both community membership (*P* = 0.006) and community structure (*P* = 0.02) between FIV-infected and FIV-uninfected cats. Significant differences were also noted by AMOVA for both the Yue & Clayton (*P* = 0.005) and Jaccard (*P* < 0.001) index data. Separation of the two groups, with greater variability in membership in the FIV-infected group, was also seen with PCoA (Figure 3).

Forty significant indicator OTUs were identified (Table 5). Eleven of 22 (50%) indicator OTUs for FIV-infected cats were Proteobacteria, compared to zero of the 18 FIV-uninfected indicators (*P <* 0.01).

**Table 5:** **Indicator OTUs for FIV-infected (** ***n*** **= 19) and FIV-uninfected (** ***n*** **= 13) cats**

**FIV-infected**	**FIV-uninfected**
Unclassified Pseudomonadaceae (3 OTUs)	5 genus incertae sedis (6 OTUs)
*Staphylococcus* (2 OTUs)	*Treponema* (3 OTUs)
*Anaerobiospirillum (2 OTUs)*	Unclassified bacterium (3 OTUs)
*Elsenella*	Unclassified Lachnospiraceae (2 OTUs)
*Phenylobacterium*	*Streptococcus*
Unclassified Aerococcaceae	*Pseudobutyrivibrio*
*Pseudomonas*	*Saccharofermentans*
Unclassified Bacillaceae	Unclassified Ruminococcaceae
*Bifidobacterium*	
*Bradyrhizobium*	
*Akkermansia*	
*Cellulosimicrobium*	
*Thermus*	
*Prevotella*	
*Mezorhizobium*	
*Carnobacterium*	
Unclassified Pasteurellaceae	
*Succinivibrio*	

One OTU, *Pseudomonas*, was found in all FIV-infected cats at a relative abundance of at least 1%. *Staphylococcus* was found in 15/19 (79%) FIV-infected cats, while *Escherichia/Shigella* was found in 14/19 (74%) at that minimum relative abundance. In contrast, no OTUs were found at a relative abundance of at least 1% in any of the FIV-uninfected samples. *Pseudomonas* was found in 8/13 (62%) FIV-uninfected samples and was the only OTU found in at least 50% of samples at a relative abundance of 1% of greater.

### Oral microbiota

There were 1 430 615 sequences from 33 samples (19 FIV-infected and 14 FIV-uninfected cats) that entered the final analysis, with sequence numbers ranging from 5587 to 94 647 per sample (mean 43 352, median 38 922, SD 24 725).

**Figure 4:**
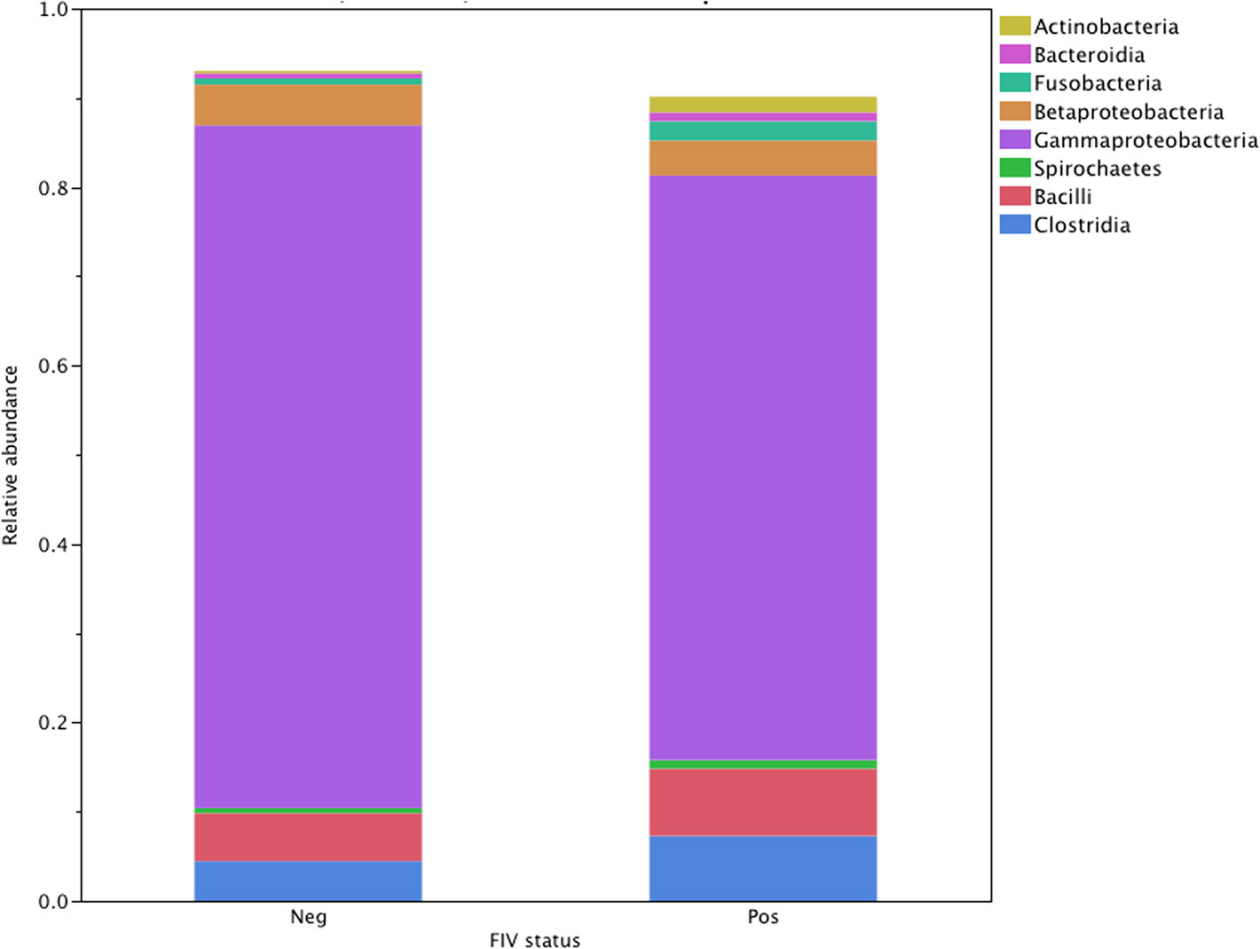
**Predominant classes in the oral microbiota of cats with and without FIV infection.** Relative abundance of the predominant classes in the oral microbiota of FIV-infected (*n* = 19) and FIV-uninfected (*n* = 14) cats. Legend: Neg = FIV-uninfected. Pos = FIV-infected. Among these predominant phyla, there were significant differences in the relative abundances for Fusobacteria (0.022 vs 0.007, *P* = 0.006) and Actinobacteria (0.017 vs 0.003, *P* = 0.003), both higher in the FIV-infected group.

Eighteen different phyla were identified (Table 6). FIV-infected cats had significantly higher relative abundances of Fusobacteria and Actinobacteria. Similarly, FIV-infected cats had significantly higher relative abundances of several bacterial classes including Fusobacteria (0.022 vs 0.007, *P* = 0.006), Actinobacteria (0.017 vs 0.003, *P* = 0.003), Sphingobacteria (0.00015 vs 0.00003, *P* = 0.0013) and Flavobacteria (0.0073 vs 0.0034, *P* = 0.030). The eight most common classes are presented in Figure 4. The predominant genera in each group are listed in Table 7, with genera that were significantly different between the two groups in Table 8.

**Table 6:** **Relative abundance of phyla in the oral cavity of FIV-infected (** ***n*** **= 19) and FIV-uninfected (** ***n*** **= 14) cats**

**Phylum**	**FIV-infected**	**FIV-uninfected**	***P*** **value**
Acidobacteria	0.000026	0.000026	0.63
Actinobacteria	0.017	0.0035	0.0097
Bacteroidetes	0.019	0.012	0.19
Chlamydiae	0.000003	0.000028	0.65
Deferribacteres	0.000002	0.000045	0.34
Deinococcus-Thermus	0.000057	0.000039	0.15
Fibrobacteres	0.0030	0.0023	0.83
Firmicutes	0.17	0.12	0.42
Fusobacteria	0.022	0.0079	0.016
Lentisphaerae	0.00016	0.00010	0.74
Planctomycetes	0.0000014	0.0000008	0.86
Proteobacteria	0.70	0.81	0.26
Spirochaetes	0.0094	0.0064	0.86
SR1	0.0019	0.0030	0.76
Synergistetes	0.000006	0.0000009	0.69
Tenericutes	0.00041	0.00040	0.28
TM7	0.00093	0.00034	0.09
Verrucomicrobia	0.0028	0.0028	0.40

**Table 7:** **Predominant genera in the oral microbiota of FIV-infected (** ***n*** **= 19) and FIV-uninfected (** ***n*** **= 14) cats**

**FIV-infected**		**FIV-uninfected**	
**Genus**	**Relative abundance**	**Genus**	**Relative abundance**
*Pasteurella*	0.27	*Pasteurella*	0.26
Unclassified Pasteurellaceae	0.12	Unclassified Pasteurellaceae	0.093
*Pseudomonas*	0.092	Unclassified Enterobacteriaceae	0.090
*Mannheimia*	0.060	*Acinetobacter*	0.0745
*Staphylococcus*	0.037	*Mannheimia*	0.061
Unclassified Clostridiales	0.030	*Pseudomonas*	0.057
*Acinetobacter*	0.030	*Kingella*	0.033
Unclassified bacterium	0.027	*Moraxella*	0.030
*Psychrobacter*	0.019	*Neisseria*	0.021
*Kingella*	0.018	Unclassified Clostridiales	0.020

**Table 8:** **Relative abundances of genera that were significantly different in the oral cavity of FIV-infected (** ***n*** **= 19) versus FIV-uninfected (** ***n*** **= 14) cats**

**Genus**	**FIV-infected**	**FIV-uninfected**	***P*** **value**
*Staphylococcus*	0.037	0.003	0.004
Unclassified Actinomycetales	0.012	0.0018	0.009
*Parvimonas*	0.008	0.002	0.005
*Streptobacillus*	0.0062	0.0019	0.017
*Sneathia*	0.0052	0.0003	0.0011
Unclassified Flavobacteriaceae	0.0033	0.0014	0.038
*Gemella*	0.0038	0.00022	0.000e
Unclassified Leptotrichiaceae	0.0038	0.0009	0.0012
*Peptoniphilus*	0.00014	0.00067	0.022

A subsample of 5587 sequences per sample was used for subsequent analysis. There were no differences in coverage (*P* = 0.64), diversity (*P* = 0.44), evenness (*P* = 0.64) or richness (*P* = 0.61) between FIV-infected and FIV-uninfected cats.

There were significant differences in microbial community structure (Yue and Clayton index, *P* = 0.030) but not community membership (Jaccard index, *P* = 0.18) by UniFrac. In contrast, there was also a significant difference between groups by AMOVA applied to a phylip-formatted distance matrix based on the Jaccard index (*P* = 0.005) but not the Yue and Clayton index (*P* = 0.078).

No OTUs were present in all cats in either the FIV-infected or FIV-uninfected groups at a minimum relative abundance of 1%. One OTU, classified as *Pseudomonas*, was present at that minimum relative abundance in 15/20 FIV-infected cats. Two OTUs, an unclassified Aerococcaceae and *Pasteurella*, were present at that abundance in 9/14 FIV-uninfected cats. Twenty indicator OTUs were identified, 16 for FIV-infected cats and four for FIV-uninfected cats (Table 9). Indicator OTUs for FIV-uninfected cats were predominantly (75%) Proteobacteria while Firmicutes were the most common indicators in FIV-uninfected cats (9/16, 56%).

**Table 9:** **Indicator OTUs for FIV-infected (** ***n*** **= 19) and FIV-uninfected (** ***n*** **= 13) cats**

**FIV-infected**	**FIV-uninfected**
Unclassified Pseudomonadaceae (3 OTUs)	5 genus incertae sedis (6 OTUs)
*Staphylococcus* (2 OTUs)	*Treponema* (3 OTUs)
*Anaerobiospirillum (2 OTUs)*	Unclassified bacterium (3 OTUs)
*Elsenella*	Unclassified Lachnospiraceae (2 OTUs)
*Phenylobacterium*	*Streptococcus*
Unclassified Aerococcaceae	*Pseudobutyrivibrio*
*Pseudomonas*	*Saccharofermentans*
Unclassified Bacillaceae	Unclassified Ruminococcaceae
*Bifidobacterium*	
*Bradyrhizobium*	
*Akkermansia*	
*Cellulosimicrobium*	
*Thermus*	
*Prevotella*	
*Mezorhizobium*	
*Carnobacterium*	
Unclassified Pasteurellaceae	
*Succinivibrio*	

**Figure 5:**
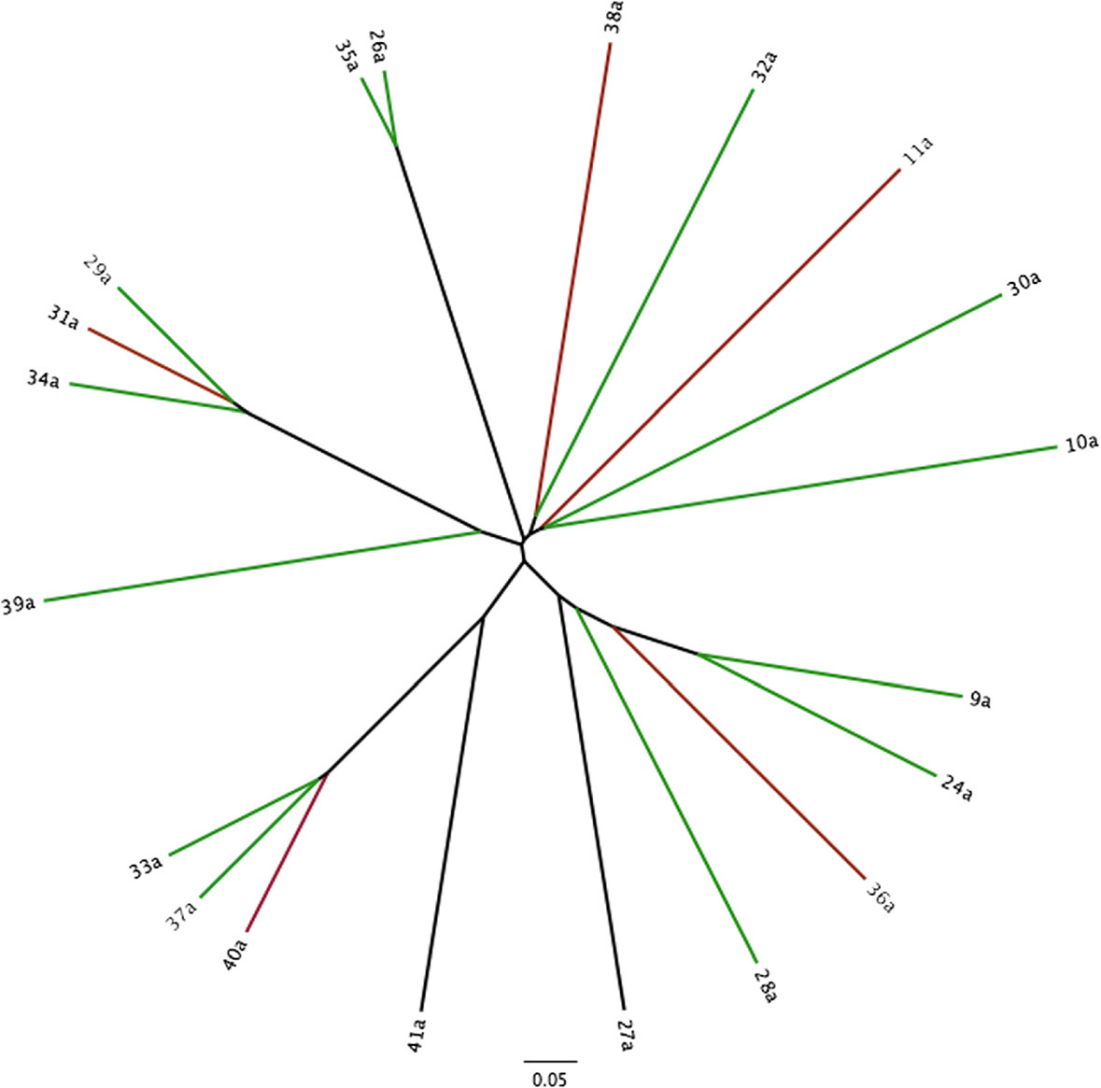
**Oral microbial community structure in cats with FIV infection.** Dendrogram based on the Yue & Clayton index of dissimilarity for the oral microbiota of 18 FIV-infected cats. Legend: Red lines indicate cats with moderate to severe gingival disease. Green lines indicate cats with mild or no gingival disease.

Four FIV infected cats had a gingival disease score of 0, nine had a score of 1, three had a score of 2 and three had a score of 3. There were no differences in community structure (*P* = 0.60) (Figure 5) or community membership (*P* = 0.99) in FIV infected cats with and without severe gingival disease (score ≥ 2). There were also no differences between the relative abundances of any phyla or classes. Similarly, there were no differences in community structure (*P* = 0.581) and membership (*P* = 0.31) in FIV-infected cats with dental disease versus those without dental disease.

## Discussion

### Conjunctival microbiota

The feline conjunctiva harbours a large number of diverse phyla, albeit at a lower richness and diversity than has been described in the feline oral cavity or intestinal tract [25-27], or identified in the oral microbiota here. Yet, while the richness and diversity were lower than reported for other mucosal surfaces, the conjunctival microbiota contained an estimate of over 1000 species per sample, a count that far exceeds culture-dependent studies [28-31].

Firmicutes dominated in FIV-uninfected cats. This broad phylum, consisting of a wide range of Gram-positive bacteria, is commonly found at most body sites, albeit with different members of the phylum predominating. For example, Clostridia are dominant Firmicutes in the intestinal tract [25,26,32], while here, Bacilli were most common. This was because of the high relative abundance of Staphylococcaceae. Staphylococci are widespread commensals of mucosal surfaces and are opportunistic pathogens that can be associated with a wide range of infections, including conjunctivitis and ulcerative keratitis [29,33,34]. Their presence in the conjunctival microbiota is not surprising based on the nature of this genus and results of previous culture-dependent studies [29,31].

Proteobacteria were also common, and at a greater relative abundance (30-37%) than in many other body sites. This phylum of Gram-negative bacteria contains a variety of potential opportunistic pathogens, including *Pseudomonas* and *Acinetobacter*, two genera that were common in clinically healthy cats.

Significant differences between FIV-infected and FIV-uninfected cats in the relative abundances of numerous phyla (and corresponding lower taxonomic levels) were identified, yet these were among lower abundance phyla, the relevance of which is unclear. The difference in relative abundance of staphylococci was striking, with a 3-fold higher abundance in the FIV-infected group. It is unusual for one genus to comprise such a large (42%) percentage of the microbiota in any body site, and the clinical relevance of this increased relative abundance in FIV-infected cats deserves further study, given the known role of staphylococci in ocular and extra-ocular disease.

Beyond comparisons of relative abundances, there are various ecological tools that can be applied to compare communities. Here, differences in both community membership and the overall microbial community structure were noted, providing more evidence of an altered microbiota community in FIV-infected cats. Another approach is indicator analysis. This is an ecological tool that identifies OTUs that “define” a community [24]. As opposed to assessment of only relative abundance, indicator analysis evaluates both relative abundance and presence or absence in a community, to identify OTUs that are strong indicators or defining characteristics of that population by their presence. Numerous indicator OTUs were identified. Interestingly, half the indicators for FIV-infected cats were Proteobacteria, compared to none for FIV-uninfected cats. While Proteobacteria were not significantly different overall between FIV-infected and FIV-uninfected cats, the predominance of genera from this phylum as indicators in FIV-infected cats suggests that some members of this genus can be specifically altered in response to FIV infection.

### Oral microbiota

Results of this study indicate differences in the microbiota based on FIV status, although results were sometimes conflicting. There were overall differences in microbial community membership and structure in some tests (e.g. UniFrac vs AMOVA) but not others. Assessing the relevance of conflicting results is difficult. The relatively small sample size could have played a role by limiting statistical power, particularly when *P* values approached significance. Differences in the study populations beyond FIV status must also be considered. While both groups were recruited from a combination of homes and a sanctuary, the differences in distribution of those sources amongst groups could also introduce some bias.

Evaluation of specific taxonomic identifications that were different between groups can be enlightening. Two phyla, Fusobacteria and Actinobacteria, were over-represented in FIV-infected cats. The significantly higher relative abundance of Fusobacteria could be relevant given the role of members of this phylum have in oral disease in other species [35]. Increased *Fusobacterium* spp has also been identified in the anal and rectal microbiotas of people with HIV [4,5]. Of additional concern is recent evidence that *F. nucleatum* can increase re-activation of HIV in vitro, indicating a possibility that increased growth of this organism could be a risk modifier for viral replication and disease progression [36]. This has not been reported with FIV and there was no difference in the abundance of *Fusobacterium* between groups (data not presented), but the notion that an altered microbiota could influence viral activation and disease progression, independent of bacterial infection, should be further investigated in cats. Actinobacteria were also over-represented in the FIV-infected group. In humans, Actinobacteria are a component of plaque [37] and are more abundant in chronic (versus acute) tooth root infections [38], but the clinical significance in cats is currently unclear.

Phylum differences were reflected in class level differences. Additionally, Sphingobacteria and Flavobacteria, both members of the phylum Bacteroidetes, were also more common in the FIV-infected group. Both are environmental organisms that can be found on body surfaces [39,40]. One could speculate that decreased local immune response could facilitate the survival of environmental organisms to which an individual is regularly exposed, but there is currently no supporting evidence for this. The statistically significant over-representation of various genera in FIV positive cats and the indicator species that were identified might also suggest that the oral cavity of this group is more amenable to colonization with organisms that are uncommon in healthy individuals, since these tended to be rare members of the community in FIV-uninfected cats.

The difference in staphylococci was interesting given the rather high relative abundance in the FIV-infected group (3.7%, compared to 0.3% in the FIV-uninfected group) and the range of infections that staphylococci can cause. Genus-level identification has limits because of the differences in virulence between different staphylococcal species. Speciation was not attempted because of the very limited species resolution of 16S rRNA gene sequencing for this genus. Yet, the overgrowth of this genus that consists of various important opportunistic pathogens raises concerns for infection risk in these cats.

In addition to *Staphylococcus,* other indicator OTUs for the FIV-infected group are of potential health concern, such as *Porphyromonas* (various species implicated in oral disease) [41,42], *Streptococcus* (different potential opportunistic pathogens, including oral pathogens) [43], *Parvimonas* (implicated in oral infections) [44,45] and *Gemella* (component of dental plaque that has been associated with opportunistic infections) [46,47].

The role of FIV infection in feline oral diseases such as gingivitis and stomatitis in cats is unclear, with some studies reporting an association between FIV infection and the prevalence or severity of oral infections [10], while others showing no association [48]. It has been suggested that the immune response to chronic antigenic stimulation or dysregulation of the immune response could be associated with increased disease risk [49] and both of those scenarios could involve the microbiota, as the microbiota is the primary antigenic exposure in the oral cavity and is known to closely interact with the immune system at other body sites [50,51]. Overgrowth of opportunistic pathogens residing in the oral cavity because of advanced immunocompromise is another potential cause.

There was no apparent impact of dental or gingival disease; however, this may be because of the small sample sizes of these subpopulations and the likely multifactorial nature of those diseases. Further, oral swabs provide an overview of the oral cavity but each swab is a composite swab of multiple different ecological niches that are present in the mouth. Therefore, changes in one specific location (e.g. plaque) might not be detected. The likelihood that FIV infection would predispose to oral infections also presumably relates in large part to the degree of immunosuppression. Most of the FIV-infected cats had clinical abnormalities, but the degree of immunocompromise was presumably variable and was not specifically studied. Study of cats with “feline AIDS” might provide more insight into changes associated with advanced immunocompromise.

### Conclusions

The feline oral and conjunctival bacterial microbiotas are diverse microbial communities consisting of hundreds or thousands of different species, but with relatively few genera predominating. Feline immunodeficiency virus infection appears to have an impact on these microbiotas. The reasons for the apparent impact of FIV infection on these external microbial communities and the clinical relevance of altered microbiotas require further study.

## Endnotes

^a^FIV/FeLV SNAP combo test, IDEXX Laboratories, Westbrook, ME, USA.

^b^IDEXX FIV realPCR test, IDEXX Laboratories, West Sacramento, CA, USA.

^c^University of Glasgow Retrovirus Research Laboratory, Glasgow, UK.

^d^E.Z.N.A. Stool DNA Kit, Omega Bio-Tek Inc., Doraville, Georgia, USA.

^e^Illumina, San Diego, USA.
